# Supplementary material for: Duration of obesity exposure between ages 10 and 40 years and its relationship with cardiometabolic disease risk factors: A cohort study
Source: PLoS Med. 2020 Dec 8;17(12):e1003387. doi: 10.1371/journal.pmed.1003387 (PMC7723271; doi:10.1371/journal.pmed.1003387)
Supplement: S1 Text — (DOCX) [file pmed.1003387.s002.docx]

**S1 Text:** **Measurement protocol for collection of cardiometabolic outcomes in adulthood**

NSHD biomedical sweep at 53 years

*Blood pressure:*

Two measures of systolic and diastolic blood pressure were taken with the participant seated and after 5 minutes of rest, using an OMRON HEM-705 (Omron, Tokyo, Japan).

*Blood biomarkers:*

50ml non-fasting blood sample was collected.

Precipitation for measurement of high-density lipoprotein (HDL) cholesterol was carried out using phosphotungstic Mg2+ on Bayer DAX-72 (Bayer AG, Leverkusen, Germany)

Samples were analyzed for HbA1c with the Tosoh A1C 2.2 Plus Analyzer (Tosoh, Tokyo, Japan) using ion exchange high performance liquid chromatography.

NCDS biomedical sweep at 44-46 years

*Blood pressure:*

Measured 3 times with participants seated and rested for 5 minutes, with an Omron 705CP automated sphygmomanometer (Omron, Tokyo, Japan), using a large cuff when mid-upper arm circumference was greater than 32 cm.

*Blood biomarkers:*

Venous blood samples were obtained without prior fasting.

HDL cholesterol was measured by Olympus model AU640 autoanalyser (Olympus Corporation, Tokyo, Japan) (Enzymatic colorimetric CHOD-PAP method).

Glycosylated haemoglobin (HbA1c) was measured on whole citrated blood by ion exchange high performance liquid chromatography, using the Tosoh A1c 2.2 Glycohemoglobin Analyser HLC-723GHb (Tosoh Corp, Tokyo, Japan).

BCS biomedical sweep at 46 years

*Blood pressure:*

Measured 3 times with participants seated and rested for 5 minutes, with an Omron HEM 907 automated sphygmomanometer (Omron, Tokyo, Japan).

*Blood biomarkers:*

Venous blood samples were obtained without prior fasting.

HDL cholesterol was measured by Roche Cobas c702 (Roche Holding AG, Basel, Switzerland) (Enzymatic colorimetric: dextran sulphate/PEG-cholesterol esterase/PEG-cholesterol oxidase/peroxidase method).

Glycosylated haemoglobin (HbA1c) was measured on whole citrated blood by ion exchange high performance liquid chromatography, using the Tosoh G8 Glycohemoglobin Analyzer (Tosoh Corp, Tokyo, Japan).
