# Supplementary material for: Duration of obesity exposure between ages 10 and 40 years and its relationship with cardiometabolic disease risk factors: A cohort study
Source: PLoS Med. 2020 Dec 8;17(12):e1003387. doi: 10.1371/journal.pmed.1003387 (PMC7723271; doi:10.1371/journal.pmed.1003387)
Supplement: S2 Text — (DOCX) [file pmed.1003387.s003.docx]

**S2 Text:** **Childhood social class**

The Registrar General’s Social Classes schema was used classify childhood social class and resulted in six social class groups: I (professional), II (managerial and technical), IIIN (skilled nonmanual), IIIM (skilled manual), IV (partly skilled), V (unskilled). The 1990 classification was used for childhood social class in the NCDS and BCS70 cohorts, whereas the 1970 version was used for childhood social class in the NSHD cohort. Those in the armed forces and not employed were not assigned a social class.
