# Supplementary material for: Duration of obesity exposure between ages 10 and 40 years and its relationship with cardiometabolic disease risk factors: A cohort study
Source: PLoS Med. 2020 Dec 8;17(12):e1003387. doi: 10.1371/journal.pmed.1003387 (PMC7723271; doi:10.1371/journal.pmed.1003387)
Supplement: S1 Table — (DOCX) [file pmed.1003387.s004.docx]

**Supplementary table S1.** **Association between ever obese and categories of obesity duration (vs never obese) and cardiometabolic disease risk factors*† (imputed, unadjusted)**

|  | **Systolic blood pressure (n=20746)** | | **Diastolic blood pressure (n=20746)** | | **HDL-cholesterol**  **(n=20746)** | | **HbA1c**  **(n=20746)** | |
| --- | --- | --- | --- | --- | --- | --- | --- | --- |
|  | n | β (95% CI) | n | β (95% CI) | n | β (95% CI) | n | β (95% CI) |
|  | *Model 1* | | | | | | | |
| Obese |  | |  | |  | |  | |
| *Never (ref)* | 17841 | - | 17841 | - | 17841 | - | 17841 | - |
| Yes | 2905 | 5.0 (4.4, 5.5) | 2905 | 5.9 (5.4, 6.5) | 2905 | -17.9 (-19.1, -16.7) | 2905 | 8.9 (8.1, 9.8) |
|  | *Model 2* | | | | | | | |
| Obesity duration |  |  |  |  |  |  |  |  |
| *Never (ref)* | 17841 | - | 17841 | - | 17841 | - | 17841 | - |
| <5 years | 757 | 4.7 (3.7, 5.7) | 757 | 5.5 (4.5, 6.6) | 757 | -13.4 (-15.5, -11.3) | 757 | 4.7 (3.4, 6.1) |
| 5-<10 years | 842 | 4.7 (3.8, 5.6) | 842 | 5.5 (4.5, 6.4) | 842 | -17.4 (-19.4, -15.4) | 842 | 6.6 (5.4, 7.8) |
| 10-<15 years | 643 | 5.2 (4.1, 6.3) | 643 | 6.6 (5.4, 7.7) | 643 | -19.2 (-21.5, -16.8) | 643 | 9.6 (7.9, 11.2) |
| 15-<20 years | 449 | 5.1 (3.8, 6.4) | 449 | 6.7 (5.3, 8.0) | 449 | -22.2 (-25.1, -19.3) | 449 | 14.5(12.4, 16.7) |
| 20-<30 years | 214 | 5.9 (4.0, 7.8) | 214 | 5.9 (3.9, 7.9) | 214 | -22.8, (-27.2, -18.5) | 214 | 19.5 (16.1, 22.8) |
| *p(trend)* |  | 0.259 |  | 0.174 |  | <0.001 |  | <0.001 |

*Values adjusted for medication use; †coefficients are on the 100 log_e_ scale, with resulting estimates expressed as symmetric percentage differences
