# Supplementary material for: Duration of obesity exposure between ages 10 and 40 years and its relationship with cardiometabolic disease risk factors: A cohort study
Source: PLoS Med. 2020 Dec 8;17(12):e1003387. doi: 10.1371/journal.pmed.1003387 (PMC7723271; doi:10.1371/journal.pmed.1003387)
Supplement: S3 Table — (DOCX) [file pmed.1003387.s006.docx]

**Supplementary table S3.** **Association between ever obese and categories of obesity duration (vs never obese) and cardiometabolic disease risk factors*† (imputed, adjusted for sex, cohort, age at follow-up, ethnicity, birth weight and childhood social class)**

|  | **Systolic blood pressure (n=20746)** | | **Diastolic blood pressure (n=20746)** | | **HDL-cholesterol**  **(n=20746)** | | **HbA1c**  **(n=20746)** | |
| --- | --- | --- | --- | --- | --- | --- | --- | --- |
|  | n | β (95% CI) | n | β (95% CI) | n | β (95% CI) | n | β (95% CI) |
|  | *Model 1* | | | | | | | |
| Obese |  | |  | |  | |  | |
| *Never (ref)* | 17841 | - | 17841 | - | 17841 | - | 17841 | - |
| Yes | 2905 | 6.1 (5.6, 6.6) | 2905 | 7.1 (6.6, 7.7) | 2905 | -16.4 (-17.6, -15.2) | 2905 | 9.0 (8.2, 9.9) |
|  | *Model 2* | | | | | | | |
| Obesity duration |  |  |  |  |  |  |  |  |
| *Never (ref)* | 17841 | - | 17841 | - | 17841 | - | 17841 | - |
| <5 years | 757 | 5.0 (4.1, 6.0) | 757 | 5.9 (4.9, 6.9) | 757 | -12.4 (-14.4, -10.4) | 757 | 4.8 (3.5, 6.2) |
| 5-<10 years | 842 | 5.7 (4.8, 6.6) | 842 | 6.6 (5.6, 7.6) | 842 | -14.8 (-16.8, -12.9) | 842 | 6.5 (5.3, 7.7) |
| 10-<15 years | 643 | 6.1 (5.1, 7.2) | 643 | 7.7 (6.6, 8.8) | 643 | -16.6 (-18.9, -14.3) | 643 | 9.5 (7.9, 11.2) |
| 15-<20 years | 449 | 7.1 (5.8, 8.4) | 449 | 8.6 (7.3, 9.9) | 449 | -21.9 (-24.7, -19.0) | 449 | 15.1 (13.0, 17.3) |
| 20-<30 years | 214 | 9.0 (7.1, 10.9) | 214 | 8.8 (6.7, 11.0) | 214 | -24.8 (-29.1, -20.5) | 214 | 19.9 (16.5, 23.3) |
| *p(trend)* |  | <0.001 |  | 0.002 |  | <0.001 |  | <0.001 |

*Values adjusted for medication use; †coefficients are on the 100 log_e_ scale, with resulting estimates expressed as symmetric percentage differences
