# Supplementary material for: Duration of obesity exposure between ages 10 and 40 years and its relationship with cardiometabolic disease risk factors: A cohort study
Source: PLoS Med. 2020 Dec 8;17(12):e1003387. doi: 10.1371/journal.pmed.1003387 (PMC7723271; doi:10.1371/journal.pmed.1003387)
Supplement: S5 Table — (DOCX) [file pmed.1003387.s008.docx]

**Supplementary table S5.** **Association between ever obese and categories of obesity duration (vs never obese) and cardiometabolic disease risk factors*† (imputed, adjusted for sex, cohort, age at follow-up, ethnicity, birth weight, childhood social class and obesity severity)**

|  | **Systolic blood pressure (n=20746)** | | **Diastolic blood pressure (n=20746)** | | **HDL-cholesterol**  **(n=20746)** | | **HbA1c**  **(n=20746)** | |
| --- | --- | --- | --- | --- | --- | --- | --- | --- |
|  | n | β (95% CI) | n | β (95% CI) | n | β (95% CI) | n | β (95% CI) |
|  | *Model 1* | | | | | | | |
| Obese |  | |  | |  | |  | |
| *Never (ref)* | 17841 | - | 17841 | - | 17841 | - | 17841 | - |
| Yes | 2905 | 4.8 (4.1, 5.5) | 2905 | 5.6 (4.9, 6.3) | 2905 | -12.3 (-13.8, -10.8) | 2905 | 4.5 (3.5, 5.6) |
|  | *Model 2* | | | | | | | |
| Obesity duration |  |  |  |  |  |  |  |  |
| *Never (ref)* | 17841 | - | 17841 | - | 17841 | - | 17841 | - |
| <5 years | 757 | 4.8 (3.9, 5.8) | 757 | 5.5 (4.5, 6.6) | 757 | -11.9 (-13.9, -9.9) | 757 | 4.4 (3.1, 5.7) |
| 5-<10 years | 842 | 4.9 (3.9, 5.9) | 842 | 5.4 (4.3, 6.4) | 842 | -13.0 (-15.1, -10.9) | 842 | 4.9 (3.4, 6.3) |
| 10-<15 years | 643 | 4.6 (3.2, 6.0) | 643 | 5.2 (3.7, 6.7) | 643 | -12.9 (-16.0, -9.8) | 643 | 6.3 (4.0, 8.6) |
| 15-<20 years | 449 | 4.5 (2.3, 6.6) | 449 | 4.2 (2.1, 6.4) | 449 | -15.5 (-20.1, -10.9) | 449 | 9.6 (6.0, 13.2) |
| 20-<30 years | 214 | 517 (1.9, 8.2) | 214 | 2.2 (-1.15, 5.6) | 214 | -15.2 (-22.4, -8.0) | 214 | 11.6 (5.9, 17.2) |
| *p(trend)* |  | 0.975 |  | 0.294 |  | 0.117 |  | 0.007 |

*Values adjusted for medication use; †coefficients are on the 100 log_e_ scale, with resulting estimates expressed as symmetric percentage differences
