# Supplementary material for: Duration of obesity exposure between ages 10 and 40 years and its relationship with cardiometabolic disease risk factors: A cohort study
Source: PLoS Med. 2020 Dec 8;17(12):e1003387. doi: 10.1371/journal.pmed.1003387 (PMC7723271; doi:10.1371/journal.pmed.1003387)
Supplement: S6 Table — (DOCX) [file pmed.1003387.s009.docx]

**Supplementary table S6.** **Association between ever obese and categories of obesity duration (vs never obese) and dichotomous cardiometabolic outcomes (imputed, adjusted for sex, cohort, age at follow-up, ethnicity, birth weight, childhood social class and obesity severity)**

|  | **Hypertension^a^**  **(n=20746)**  *(ref=normotensive)* | | | **Low HDL-cholesterol^b^**  **(n=20746)**  *(ref=non-low)* | | | **Elevated HbA1c^c^**  **(n=20746)**  *(ref=non-elevated)* | | |
| --- | --- | --- | --- | --- | --- | --- | --- | --- | --- |
|  | No | Yes | RR  (95% CI) | No | Yes | RR  (95% CI) | No | Yes | RR  (95% CI) |
|  | *Model 1* | | | | | | | | |
| Obese |  | | |  | | |  | | |
| *Never (ref)* | 12675 | 5166 | - | 15378 | 2463 | - | 15609 | 2232 | - |
| Yes | 1569 | 1336 | 1.6 (1.5, 1.7) | 1877 | 1028 | 2.0 (1.8, 2.2) | 1954 | 951 | 2.2 (2.0, 2.4) |
|  | *Model 2* | | | | | | | | |
| Obesity duration |  | |  |  | |  |  | |  |
| *Never (ref)* | 12675 | 5166 | - | 15378 | 2463 | - | 15609 | 2232 | - |
| <5 years | 440 | 317 | 1.5 (1.4, 1.6) | 537 | 220 | 2.0 (1.7, 2.3) | 569 | 188 | 2.0 (1.8, 2.3) |
| 5-<10 years | 466 | 376 | 1.7 (1.5, 1.8) | 562 | 280 | 2.0 (1.8, 2.3) | 594 | 248 | 2.3 (2.0, 2.6) |
| 10-<15 years | 339 | 304 | 1.6 (1.4, 1.8) | 412 | 231 | 2.1 (1.8, 2.5) | 428 | 215 | 2.4 (2.1, 2.8) |
| 15-<20 years | 229 | 220 | 1.6 (1.3, 1.9) | 255 | 194 | 2.5 (2.1, 3.0) | 259 | 190 | 3.0 (2.4, 3.7) |
| 20-<30 years | 95 | 119 | 1.6 (1.3, 2.1) | 111 | 103 | 2.6 (2.0, 3.4) | 104 | 110 | 3.0 (2.3, 4.0) |
| *p(trend)* |  |  | 0.456 |  |  | 0.037 |  |  | 0.006 |

^a^Hypertension: SBP/DBP≥140/90mmHg and/or on BP lowering medication; ^b^Low-HDL: according to NCEP ATPIII criteria and/or on lipid-regulating medication; ^c^Elevated HbA1c: according to CDC criteria and/or on diabetes medication
