# Supplementary material for: Duration of obesity exposure between ages 10 and 40 years and its relationship with cardiometabolic disease risk factors: A cohort study
Source: PLoS Med. 2020 Dec 8;17(12):e1003387. doi: 10.1371/journal.pmed.1003387 (PMC7723271; doi:10.1371/journal.pmed.1003387)
Supplement: S7 Table — (DOCX) [file pmed.1003387.s010.docx]

**Supplementary table S7.** **Association between ever obese and categories of obesity duration (vs never obese) and cardiometabolic disease risk factors (imputed, adjusted for sex, cohort, age at follow-up, ethnicity, birth weight, childhood social class and obesity severity): excluding NSHD**

|  | **Systolic blood pressure (n=17778)** | | **Diastolic blood pressure (n=17778)** | | **HDL-cholesterol**  **(n=17778)** | | **HbA1c**  **(n=17778)** | |
| --- | --- | --- | --- | --- | --- | --- | --- | --- |
|  | n | β (95% CI) | n | β (95% CI) | n | β (95% CI) | n | β (95% CI) |
|  | *Model 1* | | | | | | | |
| Obese |  | |  | |  | |  | |
| *Never (ref)* | 15070 | - | 15070 | - | 15070 | - | 15070 | - |
| Yes | 2708 | 4.8 (4.1, 5.5) | 2708 | 5.8 (5.0, 6.5) | 2708 | -12.1 (-13.6, -10.5) | 2708 | 4.4 (3.3, 5.4) |
|  | *Model 2* | | | | | | | |
| Obesity duration |  |  |  |  |  |  |  |  |
| *Never (ref)* | 15070 | - | 15070 | - | 15070 | - | 15070 | - |
| <5 years | 668 | 4.7 (3.7, 5.6) | 668 | 5.7 (4.6, 6.8) | 668 | -11.4 (-13.5, -9.3) | 668 | 4.2 (2.9, 5.6) |
| 5-<10 years | 799 | 4.9 (3.9, 6.0) | 799 | 5.5 (4.4, 6.7) | 799 | -12.7 (-14.8, -10.5) | 799 | 4.7 (3.2, 6.2) |
| 10-<15 years | 609 | 4.8 (3.4, 6.3) | 609 | 5.4 (3.9, 7.0) | 609 | -13.1 (-16.3, -10.0) | 609 | 6.1 (3.7, 8.5) |
| 15-<20 years | 430 | 4.5 (2.3, 6.7) | 430 | 4.5 (2.2, 6.7) | 430 | -15.9 (-20.5, -11.3) | 430 | 9.5 (5.8, 13.2) |
| 20-<30 years | 202 | 5.0 (1.9, 8.2) | 202 | 3.0 (-0.5, 6.4) | 202 | -15.4 (-22.5, -8.3) | 202 | 11.3 (5.3, 17.2) |
| *p(trend)* |  | 0.830 |  | 0.451 |  | 0.070 |  | 0.010 |

*Values adjusted for medication use; †coefficients are on the 100 log_e_ scale, with resulting estimates expressed as symmetric percentage differences
