# Supplementary material for: Duration of obesity exposure between ages 10 and 40 years and its relationship with cardiometabolic disease risk factors: A cohort study
Source: PLoS Med. 2020 Dec 8;17(12):e1003387. doi: 10.1371/journal.pmed.1003387 (PMC7723271; doi:10.1371/journal.pmed.1003387)
Supplement: S8 Table — (DOCX) [file pmed.1003387.s011.docx]

**Supplementary table S8.** **Association between ever obese and categories of obesity duration (vs never obese) and dichotomous cardiometabolic outcomes (imputed, adjusted for sex, cohort, age at follow-up, ethnicity, birth weight, childhood social class and obesity severity): excluding NSHD**

|  | **Hypertension^a^**  **(n=17778)**  *(ref=normotensive)* | | | **Low HDL-cholesterol^b^**  **(n=17778)**  *(ref=non-low)* | | | **Elevated HbA1c^c^**  **(n=17778)**  *(ref=non-elevated)* | | |
| --- | --- | --- | --- | --- | --- | --- | --- | --- | --- |
|  | No | Yes | RR  (95% CI) | No | Yes | RR  (95% CI) | No | Yes | RR  (95% CI) |
|  | *Model 1* | | | | | | | | |
| Obese |  | | |  | | |  | | |
| *Never (ref)* | 11751 | 3319 | - | 12921 | 2149 | - | 13787 | 1283 | - |
| Yes | 1527 | 1181 | 1.7 (1.6, 1.8) | 1740 | 968 | 2.0 (1.8, 2.2) | 1867 | 841 | 2.5 (2.3, 2.8) |
|  | *Model 2* | | | | | | | | |
| Obesity duration |  | |  |  | |  |  | |  |
| *Never (ref)* | 11751 | 3319 | - | 12921 | 2149 | - | 13787 | 1283 | - |
| <5 years | 417 | 251 | 1.7 (1.5 1.8) | 474 | 194 | 1.9 (1.7, 2.2) | 526 | 142 | 2.3 (1.9, 2.8) |
| 5-<10 years | 461 | 338 | 1.8 (1.6, 2.0) | 535 | 264 | 2.0 (1.7, 2.3) | 576 | 223 | 2.6 (2.2, 3.0) |
| 10-<15 years | 334 | 275 | 1.8 (1.6, 2.0) | 388 | 221 | 2.1 (1.8, 2.5) | 415 | 194 | 2.8 (2.3, 3.3) |
| 15-<20 years | 224 | 206 | 1.8 (1.5, 2.6) | 241 | 189 | 2.5 (2,1, 3.1) | 249 | 181 | 3.5 (2.8, 4.3) |
| 20-<30 years | 91 | 111 | 2.0 (1.5, 2.9) | 102 | 100 | 2.6 (2.0, 3.5) | 101 | 101 | 3.6 (2.6, 4.9) |
| *p(trend)* |  |  | 0.228 |  |  | 0.025 |  |  | 0.004 |

^a^Hypertension: SBP/DBP≥140/90mmHg and/or on BP lowering medication; ^b^Low-HDL: according to NCEP ATPIII criteria and/or on lipid-regulating medication; ^c^Elevated HbA1c: according to CDC criteria and/or on diabetes medication
