# Supplementary material for: Duration of obesity exposure between ages 10 and 40 years and its relationship with cardiometabolic disease risk factors: A cohort study
Source: PLoS Med. 2020 Dec 8;17(12):e1003387. doi: 10.1371/journal.pmed.1003387 (PMC7723271; doi:10.1371/journal.pmed.1003387)
Supplement: S9 Table — (DOCX) [file pmed.1003387.s012.docx]

**Supplementary table S9.** **Association between ever obese and categories of obesity duration (vs never obese) and cardiometabolic disease risk factors (imputed, adjusted for sex, cohort, age at follow-up, ethnicity, birth weight, childhood social class and obesity severity): using blood pressure at 43 years in NSHD**

|  | **Systolic blood pressure (n=20746)** | | **Diastolic blood pressure (n=20746)** | |
| --- | --- | --- | --- | --- |
|  | n | β (95% CI) | n | β (95% CI) |
|  | *Model 1* | | | |
| Obese |  | |  | |
| *Never (ref)* | 17841 | - | 17841 | - |
| Yes | 2905 | 4.7 (4.0, 5.4) | 2905 | 5.6 (4.9; 6.4) |
|  | *Model 2* | | | |
| Obesity duration |  | |  | |
| *Never (ref)* | 17841 | - | 17841 | - |
| <5 years | 757 | 4.5 (3.6, 5.5) | 757 | 5.6 (4.5, 6.6) |
| 5-<10 years | 842 | 4.9 (3.9, 5.9) | 842 | 5.4 (4.3, 6.6) |
| 10-<15 years | 643 | 4.8 (3.4, 6.2) | 643 | 5.1 (3.6, 6.6) |
| 15-<20 years | 449 | 4.6 (2.5, 6.6) | 449 | 4.1 (1.9, 6.4) |
| 20-<30 years | 214 | 5.0 (1.9, 8.0) | 214 | 2.4 (-1.0, 5.7) |
| *p(trend)* |  | 0.782 |  | 0.264 |

*Values adjusted for medication use; †coefficients are on the 100 log_e_ scale, with resulting estimates expressed as symmetric percentage differences
