# Supplementary material for: Duration of obesity exposure between ages 10 and 40 years and its relationship with cardiometabolic disease risk factors: A cohort study
Source: PLoS Med. 2020 Dec 8;17(12):e1003387. doi: 10.1371/journal.pmed.1003387 (PMC7723271; doi:10.1371/journal.pmed.1003387)
Supplement: S10 Table — (DOCX) [file pmed.1003387.s013.docx]

**Supplementary table S10.** **Association between ever obese and categories of obesity duration (vs never obese) and categorical cardiometabolic outcomes (imputed, adjusted for sex, cohort, age at follow-up, ethnicity, birth weight, childhood social class and obesity severity): using blood pressure at 43 years in NSHD**

|  | **Hypertension^a^**  **(n=20746)** | | |
| --- | --- | --- | --- |
|  | No | Yes | RR (95% CI) |
| Obese |  | | |
| *Never (ref)* | 12620 | 5221 | - |
| Yes | 1571 | 1334 | 1.6 (1.5, 1.7) |
|  |  | |  |
| Obese |  | | |
| *Never (ref)* | 12620 | 5221 | - |
| <5 years | 441 | 316 | 1.5 (1.4, 1.6) |
| 5-<10 years | 466 | 376 | 1.6 (1.5, 1.8) |
| 10-<15 years | 339 | 304 | 1.6 (1.4, 1.8) |
| 15-<20 years | 229 | 220 | 1.6 (1.3, 1.9) |
| 20-<30 years | 96 | 118 | 1.6 (1.3, 2.1) |
| *p(trend)* |  |  | 0.460 |

^a^Hypertension: SBP/DBP≥140/90mmHg and/or on BP lowering medication
