# Supplementary material for: Duration of obesity exposure between ages 10 and 40 years and its relationship with cardiometabolic disease risk factors: A cohort study
Source: PLoS Med. 2020 Dec 8;17(12):e1003387. doi: 10.1371/journal.pmed.1003387 (PMC7723271; doi:10.1371/journal.pmed.1003387)
Supplement: S11 Table — (DOCX) [file pmed.1003387.s014.docx]

**Supplementary table S11.** **Association between ever obese and categories of obesity duration (vs never obese) and cardiometabolic disease risk factors (imputed, adjusted for cohort, age at follow-up, ethnicity, birth weight, childhood social class and obesity severity): sex interaction**

|  | **Systolic blood pressure (n=20746)** | | **Diastolic blood pressure (n=20746)** | | **HDL-cholesterol**  **(n=20746)** | | **HbA1c**  **(n=20746)** | |
| --- | --- | --- | --- | --- | --- | --- | --- | --- |
|  | n | β (95% CI) | n | β (95% CI) | n | β (95% CI) | n | β (95% CI) |
|  | *Model 1* | | | | | | | |
| Obese (*males=ref)*) |  | |  | |  | |  | |
| Females | 1441 | 2.1 (1.1, 3.1) | 1441 | 3.3 (2.2, 4.4) | 1441 | -6.9 (-9.2, -4.5) | 1441 | -2.0 (-3.6, 0.5) |
| *p(interaction)* |  | p<0.001 |  | p<0.001 |  | p<0.001 |  | p=0.011 |
|  | *Model 2* | | | | | | | |
| Obesity duration (*males= ref*) |  |  |  |  |  |  |  |  |
| <5 years | 372 | 1.5 (-0.4, 3.3) | 372 | 1.7 (-.3, 3.8) | 372 | -9.5 (-13.6, -5.4) | 372 | 0.4 (-2.1, 3.0) |
| 5-<10 years | 384 | 2.1 (0.3, 3.9) | 384 | 3.6 (1.7, 5.6) | 384 | -4.7 (-8.6, -0.7) | 384 | -2.4 (-4.8, -0.1) |
| 10-<15 years | 284 | 0.7 (-1.4, 2.8) | 284 | 2.7 (0.5, 5.0) | 284 | -7.9 (-12.5, -3.2) | 284 | 0.4 (-2.9, 3.8) |
| 15-<20 years | 253 | 3.5 (0.9, 6.1) | 253 | 4.2 (1.5, 6.9) | 253 | -5.8 (-11.2, -0.4) | 253 | -9.4 (-13.9, -4.9) |
| 20-<30 years | 148 | 6.2 (2.3, 10.1) | 148 | 8.6 (4.5, 12.6) | 148 | -6.0 (-15.1, 3.1) | 148 | -0.4 (-7.9, 7.1) |
| *p(interaction)* |  | 0.308 |  | 0.707 |  | 0.248 |  | 0.129 |

*Values adjusted for medication use; †coefficients are on the 100 log_e_ scale, with resulting estimates expressed as symmetric percentage differences
