# Supplementary material for: Duration of obesity exposure between ages 10 and 40 years and its relationship with cardiometabolic disease risk factors: A cohort study
Source: PLoS Med. 2020 Dec 8;17(12):e1003387. doi: 10.1371/journal.pmed.1003387 (PMC7723271; doi:10.1371/journal.pmed.1003387)
Supplement: S12 Table — (DOCX) [file pmed.1003387.s015.docx]

**Supplementary table S12.** **Association between ever obese and categories of obesity duration (vs never obese) and dichotomous cardiometabolic outcomes (imputed, adjusted for cohort, age at follow-up, ethnicity, birth weight, childhood social class and obesity severity): sex interaction**

|  | **Hypertension^a^**  **(n=20746)**  *(ref=normotensive)* | **Low HDL-cholesterol^b^**  **(n=20746)**  *(ref=non-low)* | **Elevated HbA1c^c^**  **(n=20746)**  *(ref=non-elevated)* |
| --- | --- | --- | --- |
|  | RR  (95% CI) | RR  (95% CI) | RR  (95% CI) |
|  |  |  |  |
| Obese (*males=ref*) |  |  |  |
| Females | 1.2 (1.1, 1.3) | 1.4 (1.2, 1.6) | 1.1 (0.97, 1.31) |
| *p(interaction)* | p=0.001 | p<0.001 | p=0.126 |
|  |  |  |  |
| Obesity duration (*males=ref*) |  |  |  |
| <5 years | 1.0 (0.9, 1.3) | 1.7 (1.3, 2.1) | 1.3 (0.93, 1.69) |
| 5-<10 years | 1.3 (1.1, 1.5) | 1.2 (0.9, 1.5) | 1.0 (0.78, 1.32) |
| 10-<15 years | 1.1 (0.9, 1.3) | 1.6 (1.3, 2.1) | 1.1 (0.87, 1.51) |
| 15-<20 years | 1.3 (1.0, 1.6) | 1.4 (1.1, 1.8) | 1.1 (0.83, 1.44) |
| 20-<30 years | 1.5 (1.1, 2.1) | 1.3 (0.9, 1.9) | 1.3 (0.91, 1.82) |
| *p(interaction)* | 0.093 | 0.071 | 0.02 |

^a^Hypertension: SBP/DBP≥140/90mmHg and/or on BP lowering medication; ^b^Low-HDL: according to NCEP ATPIII criteria and/or on lipid-regulating medication; ^c^Elevated HbA1c: according to CDC criteria and/or on diabetes medication
