# Supplementary material for: Duration of obesity exposure between ages 10 and 40 years and its relationship with cardiometabolic disease risk factors: A cohort study
Source: PLoS Med. 2020 Dec 8;17(12):e1003387. doi: 10.1371/journal.pmed.1003387 (PMC7723271; doi:10.1371/journal.pmed.1003387)
Supplement: S13 Table — (DOCX) [file pmed.1003387.s016.docx]

**Supplementary table S13.** **Association between ever obese and categories of obesity duration (vs never obese) and cardiometabolic disease risk factors (imputed, adjusted for sex, cohort, age at follow-up, ethnicity, birth weight, childhood social class and obesity severity): limited to those who once obese were always obese**

|  | **Systolic blood pressure (n=20697)** | | **Diastolic blood pressure (n=20697)** | | **HDL-cholesterol**  **(n=20697)** | | **HbA1c**  **(n=20697)** | |
| --- | --- | --- | --- | --- | --- | --- | --- | --- |
|  | n | β (95% CI) | n | β (95% CI) | n | β (95% CI) | n | β (95% CI) |
|  | *Model 1* | | | | | | | |
| Obese |  | |  | |  | |  | |
| *Never (ref)* | 17841 | - | 17841 | - | 17841 | - | 17841 | - |
| Yes | 2856 | 4.9 (4.2, 5.5) | 2856 | 5.7 (4.9, 6.4) | 2856 | -12.4 (-13.9, -10.9) | 2856 | 4.6 (3.6, 5.7) |
|  | *Model 2* | | | | | | | |
| Obesity duration |  |  |  |  |  |  |  |  |
| *Never (ref)* | 17841 | - | 17841 | - | 17841 | - | 17841 | - |
| <5 years | 739 | 4.9 (3.9, 5.8) | 739 | 5.6 (4.6, 6.7) | 739 | -12.2 (-14.2, -10.1) | 739 | 4.5 (3.2, 5.9) |
| 5-<10 years | 833 | 5.0 (4.0, 6.0) | 833 | 5.4 (4.2, 6.5) | 833 | -12.9 (-15.0, -10.7) | 833 | 4.9 (3.4, 6.4) |
| 10-<15 years | 636 | 4.8 (3.4, 6.2) | 636 | 5.2 (3.7, 6.8) | 636 | -12.7 (-15.8, -9.6) | 636 | 6.4 (4.0, 8.7) |
| 15-<20 years | 442 | 4.6 (2.5, 6.8) | 442 | 4.2 (2.0, 6.4) | 442 | -15.0 (-20.0, -10.3) | 442 | 9.8 (6.2, 13.5) |
| 20-<30 years | 206 | 5.3 (2.1, 8.5) | 206 | 2.2 (-1.3, 5.7) | 206 | -14.4 (-21.7, -7.1) | 206 | 10.5 (5.0, 16.1) |
| *p(trend)* |  | 0.852 |  | 0.301 |  | 0.220 |  | 0.011 |

*Values adjusted for medication use; †coefficients are on the 100 log_e_ scale, with resulting estimates expressed as symmetric percentage differences
