# Supplementary material for: Duration of obesity exposure between ages 10 and 40 years and its relationship with cardiometabolic disease risk factors: A cohort study
Source: PLoS Med. 2020 Dec 8;17(12):e1003387. doi: 10.1371/journal.pmed.1003387 (PMC7723271; doi:10.1371/journal.pmed.1003387)
Supplement: Attachment — Submitted filename: Response to reviewer comments_v2.docx [file pmed.1003387.s019.docx]

**Response to reviewer comments**

Editorial comments and requests

*The abstract should be modified to use the PLOS Medicine structure (Background, Methods and Findings, Conclusions) - "Methods and Findings" is a single subsection.

*Response: Done.*

*In the last sentence of the Abstract Methods and Findings section, please note any key limitation(s) of the study's methodology.

*Response: The following sentence has now been added (page 3, line 71-74): “Due to pooling of three cohorts and thus the availability of only a limited number harmonised variables across cohorts, our models included adjustment for only a small number of potential confounding variables, meaning there is a possibility of residual confounding”.*

*At this stage, we ask that you include a short, non-technical Author Summary of your research to make findings accessible to a wide audience that includes both scientists and non-scientists. The Author Summary should immediately follow the Abstract in your revised manuscript. This text is subject to editorial change and should be distinct from the scientific abstract. Please see our author guidelines for more information: https://journals.plos.org/plosmedicine/s/revising-your-manuscript#loc-author-summary

*Response: An Author Summary has been added.*

*The main manuscript text should have slightly restructured headings to fit the usual PLOS Medicine style for research articles, with the main opening section headed "Introduction" and then a "Methods" (or "Methods and Materials") section.

*Response: This has now been relabelled as ‘Introduction’ (page 5, line 113) and ‘Methods’ (page 7, line 184)*

*If possible, please reformat the in-text citation callouts to use sequential numerals in square brackets (eg, [1], [2], [3]) - if using referencing software this should be fairly straightforward and quick.

*Response: The PLOS Medicine style has now been used throughout.*

*It would be good to clarify in the paper if this analysis corresponded to an analytical plan that was prospectively planned out (eg prior to data collection/examination of data). Please state this (either way) early in the Methods section.

a) If a prospective analysis plan (from your funding proposal, IRB or other ethics committee submission, study protocol, or other planning document written before analyzing the data) was used in designing this analysis, please include the relevant prospectively written document with your revised manuscript as a Supporting Information file to be published alongside your study, and cite it in the Methods section. A legend for this file should be included at the end of your manuscript.

b) If no such document exists, please make sure that the Methods section transparently describes when analyses were planned, and when/why any data-driven changes to analyses took place.

c) In either case, changes in the analysis-- including those made in response to peer review comments-- should be identified as such in the Methods section of the paper, with rationale.

*Response: We have added the following sentence at the start of the statistical analysis sub-section (page 8, line 217): “TN and WJ determined which analyses to perform and include in the present paper in January 2019 after discussing options with all co-authors”.*

Reviewer comments:

***Reviewer #1:***

The adverse cardio-metabolic consequences are well known. In this paper, the authors made an effort to dissect the effects of obesity severity and obesity duration on four metabolic risk markers, namely diastolic blood pressure, systolic blood pressure, HDL-cholesterol and glycated haemoglobin (HbA1c). They utilized data collected in three British cohort studies including over 20,000 individuals. They found that a greater obesity duration was associated with worse values for all measured risk markers. The largest association with obesity duration was for HbA1c. When authors adjusted for obesity severity, the association with obesity duration was attenuated for blood pressure and HDL-cholesterol, but persisted for HbA1c. From this, they concluded that by reducing lifetime exposure to obesity may help to reduce risk of diabetes, independently of obesity severity.

Overall, I found this study well done. Below are some remarks that the authors may wish to consider:

*We thank the reviewer for their insightful comments and feedback. We have addressed each of the comments below.*

**1.	Line 80. "Obesity is a global public health concern, with worldwide prevalence increasing from 100 81 million in 1975 to 671 million in 2015".**

**This is could be more informative, e.g. The prevalence of obesity, defined as X, has increased from X% in 1975 to Y% in 2015 etc.**

*Response:* *We have restructured the opening two sentences, which now read: (page 5, line 114) “Obesity is a global public health concern. Worldwide prevalence of child and adolescent obesity (defined according to a BMI> 2 standard deviations above age-specific World Health Organization cut-offs) has increased from 0.9% and 0.7% in boys and girls, respectively, in 1975 to 7.8% and 5.6%, respectively, in 2016. These increases in child obesity accompany significant increases in global adult obesity, with prevalence increasing from 3% and 6.6% of males and females, respectively, in 1975 to 11.6% and 15.7%, respectively, in 2016 (defined according to a body mass index>30kg/m2).”*

**2.	If venous sample was taken, it is unclear why only HbA1c and HDL-cholesterol have been analysed. This is clearly a limitation that should be explained.**

*Response: This work was based on the pooling of data from three cohort studies which requires the collection of the same (or harmonised) variables in each of the cohorts. Accordingly, we identified an a priori set of cardiometabolic risk factors, namely SBP, DBP, HDL, triglycerides and HbA1c which were collected at the mid-adulthood biomedical sweeps in each of the cohorts and thus were able to be pooled. During the analysis process, it became apparent that numbers would be substantially reduced for analyses involving triglycerides due to data collection issues experienced in the BCS70 cohort. Therefore we removed this variable from the list of cardiometabolic risk factors, leaving us with just SBP, DBP, HDL and HbA1c.*

**3.	The authors have used the International Obesity Task Force cut-points to define obesity. They may want consider performing sensitivity analyses by using more recently published cut-points from the i3C consortium (Lancet Child Adolesc Health. 2019;3:795-802).**

*Response: Though this would be interesting to do, it would necessitate a complete re-analysis for what may result in minor changes in the relationship between obesity duration and cardiometabolic risk factors. The i3C cut-offs are lower than those of the IOTF and thus may categorise a greater number of children as obese. However, we are not sure whether this would change the relationship between obesity duration and outcomes. Our initial decision to use the IOTF cut-offs was because these are currently the most widely used and were available at the time of the analysis.*

**4.	The -5% constant for HDL-cholesterol seems quite high, as commonly used lipid lowering medications have a neglible effect on HDL-cholesterol.**

*Response: While we acknowledge that the efficacy in lipid-regulating medications is dependent on the type of drug used, we believe a constant of 5% is well within the estimates reported in meta-analyses looking at the effects of such drugs on HDL concentrations. For example, Wierzbicki et al (2002) reported effects in the region of 4.0 to 10.0% for atorvastatin 10 and 20 mg. In contrast, HDL-C levels were consistently increased across all doses of simvastatin (6.8–11.0% at 10 mg, 5.2–9.0% at 20 mg, 6.7–9.6% at 40 mg and 6.6–9.7% at 80 mg). In the meta-analysis by Costa et al (2006), HDL increased by 5-7.5% in a number of trials with various lipid-regulating drugs. More recently, the HPS2-THRIVE RCT into the effects of niacin observed average increases in HDL of 6 mg per deciliter in the intervention group, an increase in approximately 13%. We have now also added the Costa (2006) reference to the list of supporting references in order to strengthen this support.*

**5.	The authors have not considered any covariates. Should potential confounders be taken into account, such as smoking, socioeconomic status and physical activity/sedentarity?**

*Response:* *We believe that variables to be adjusted for should occur prior to the exposure and outcome and thus have the potential to confound. As such, we have adjusted for sex and cohort (and age at follow-up acting as a competing exposure). Additionally, as we are combining three birth cohorts, it is important that any variables that go into the analysis represents the same thing in each cohort (i.e. variables are harmonised across cohorts). Unfortunately, this is only the case for a number of variables in the three cohorts we have used. However, to address this comment we have re-run the analyses additionally adjusting for social class, birth weight, and ethnicity. These variables meet the necessary criteria of 1) being a potential confounder (i.e., potential to cause exposure and outcome) and 2) being measured in each cohort in the same way at the same age. This has necessitated the use of multiple imputation to account for missingness in the covariates. Appropriate detail has been added to the methods section (page 9, lines 279-287).*

***Reviewer #2:***

This is a well-analyzed and nicely written paper that will make a useful contribution to the literature. I have only a few comments:

Response: *We thank the reviewer for their suggestions as to how our work can be improved. We have addressed all of the comments below*.

1. **Why not include triglyceride level or LDL here? Including these would strengthen the paper, if they are available.**

*This work was based on the pooling of data from three cohort studies which requires the collection of the same (or harmonised) variables in each of the cohorts. Accordingly, we identified an a priori set of cardiometabolic risk factors, namely SBP, DBP, HDL, triglycerides and HbA1c which were collected at the mid-adulthood biomedical sweeps in each of the cohorts and thus were able to pooled. Whilst it would have been interesting to look at LDL in addition to HDL, unfortunately this was not collected during the biomedical sweep of the BCS70 cohort. Additionally, while triglycerides were collected in all three cohorts, it became apparent during the analysis process that numbers would be substantially reduced for analyses involving triglycerides due to data collection issues experienced in the BCS70 cohort. Therefore we removed this variable from the list of cardiometabolic risk factors, leaving us with just SBP, DBP, HDL and HbA1c.*

**2.	Figure 3. Why not show estimates for dichotomous outcomes here as well? Dichotomizing variables is generally considered less ideal, but in this case they have the benefit of not making assumptions about the impact of medication use on continuous variables, which are certain to have individual variation. This means that both analyses will contain bias and so providing both in the main figure is useful. In addition, the dichotomized variables are tied to diagnostic criteria based on risk for cardiometabolic events and may make this paper more accessible to clinicians.**

*Response: We have now added this figure as figure 4, showing the relationship between obesity duration and hypertension and low HDL.*

2. **Did you conduct formal assessment of heterogeneity by sex?**

*Response: In the modelling of serial BMI measurements to obtain life course trajectories, we incorporated sex X age interactions into the model due to the known sex differences in BMI trajectories. We have also now replaced the supplementary tables related to sex differences for a table which formally tests for this sex X obesity_duration interaction. This can be found in supplementary tables 6a and 6b.*

**4.	Be cautious conflating a non-significant p for trend with the lack of a trend, particularly when the dichotomous outcome results differ. Especially for HDL, a marginally non-significant p for trend does not mean there is no trend, it just looks messier with more uncertainty. This is particularly questionable given the highly significant and clean trend for Low HDL.**

*Response: We agree, and have revised the text to emphasise a weakening of evidence rather than a disappearance, e.g. page 12 line 368, line 374, line 384, page 13 line 388.*

**5.	A speculation from thinking about Figure 2 and 3 that may be beyond the scope of this paper: Is duration more directly predictive of severity during the early years of obesity? This would make sense given that the development of higher levels of obesity generally takes time. Then does this mean that the heterogeneity in severity develops based on something else as duration continues? This might be an additional area of investigation, but feel free to ignore it if it makes the paper unwieldy.**

*Response: We thank the reviewer for the suggestion, but believe it is beyond the scope of this paper.*

Minor points:

**1.	The continuous variables should not be labeled as "outcomes". These are cardiometabolic disease risk factors. The dichotomized variables (Hypertension, low HDL, high HbA1c) could more generally be referred to as "outcomes" since those cut-points are used for diagnostic purposes and are tied to cardiometabolic events like diabetes, CVD, and mortality. Although more precisely, "outcomes" is often reserved for these specific types of events. More appropriately, the continuous results would be labeled "Continuous Variables" and the dichotomized results would be labeled "Cardiometabolic Risk Factors".**

*Response: We have changed this throughout.*

**2.	Table 1. Providing a connection to obesity severity in a more immediately interpretable format (simple BMI) would be useful. The concept of BMI-years is not as accessible to everyone, and z-scores are notoriously hard to make intuitive and providing this in Table 1 for one of the adult assessments may help put the levels of BMI into context for readers.**

*Response: We have added a row in table 1 which documents the prevalence of obesity in each cohort at the biomedical outcome sweep.*

***Reviewer #3:***

I confine my remarks to statistical aspects of this paper.

*Response: We are grateful to the reviewer for his generous feedback and suggestions.*

**There was quite a lot to like here. The use of cubic splines to model obesity duration, for example, is excellent. But then (lines 193-196) this hard work was largely thrown away by categorizing the results. Such categorization is fine for presentation, but it weakens analysis, increasing both type I and type II error.**

**Instead of this, why not use obese years? Or use the results of the cubic spline analysis (perhaps clustering the trajectories - although that might be another paper). Or simply use the results of the trapezoid rule on lines 206-207?**

*Response: It was our a priori analysis plan to include ‘obese years’ as a continuous variable. We were unable to do so however, because it was zero inflated and severely skewed. As such, the only way we could get it into a model without violating assumptions was to categorise the variable.*

*In saying that, we did make an attempt to incorporate this variable as a continuous variable and relate it to the outcomes using fractional polynomials, via an approach suggested by Patrick Royston. In this approach, a dummy variable for obesity (yes/no) is included in the fractional polynomials relating obesity duration and each outcome variable. The model fit (BIC) is checked. In subsequent steps, we then remove the FP terms and then separately, the dummy variable, and check the change in the BIC after each model. Depending on how the model fit statistics change (or don’t change) upon removal of the dummy variable and FP terms informs us about importance of each and whether FP terms for the relationship between obesity duration should be included. When these models were ran, we then set about including adjustments for covariates, an important one being obesity severity. However, when this adjustment was included, we encountered problems with model convergence and also fitted values that resulted in an unrealistic ‘tick’ in plots of the outcomes against obesity duration. We interpreted this as model instability and took a pragmatic decision that we would instead opt to categorise the obesity duration variable as it would not only offer a potential method of identifying a non-linear relationship (acknowledging it is less optimal than a fractional polynomial), but would also make interpretation much more straightforward.*

More minor issues:

**Line 66 "largest" should probably be "strongest"**

*Response: this has now been changed (page 3, line 66).*

**Line 80-81 Please give as % of global population**

*Response: We have restructured the opening two sentences, which now read: (page 5, line 114) “Obesity is a global public health concern. Worldwide prevalence of child and adolescent obesity (defined according to a BMI> 2 standard deviations above age-specific World Health Organization cut-offs) has increased from 0.9% and 0.7% in boys and girls, respectively, in 1975 to 7.8% and 5.6%, respectively, in 2016. These increases in child obesity accompany significant increases in global adult obesity, with prevalence increasing from 3% and 6.6% of males and females, respectively, in 1975 to 11.6% and 15.7%, respectively, in 2016 (defined according to a body mass index>30kg/m2).”*

**Line 84-85 I don't see how this relates to the previous sentence. Can you explain?**

*Response: To address this comment we have rephrased as follows (page 5, line 124) “**indeed there exists a group of individuals who whilst exceeding the standard BMI cut-off for obesity (≥30 kg/m²), are regarded as metabolically healthy because they have an absence of other major cardiovascular risk factors”.*

**Line 157 What was "harmonization"? What was done and why?**

*Response: we include a table below which highlights the harmonisation process which the senior author conducted and which has now been deposited on the CLOSER website (*[*https://www.closer.ac.uk/about-the-research-we-fund/data-harmonisation/*](https://www.closer.ac.uk/about-the-research-we-fund/data-harmonisation/)*). We have also included a reference to the paper on which this process was first reported and in which the table below appears (Johnson et al 2015; doi: 10.1371/journal.pmed.1001828).*

**Line 202-203 Don't categorize independent variables.**

*Response: Please see our response to the reviewer’s first comment.*

***Reviewer #4:***

Cole et al conducted a study attempting to evaluate the independent association of overweight duration and cardiometabolic risk factors, independently of obesity severity. This is certainly an important area of investigation as an improved understanding of the independent effect of obesity duration and severity could lead to refined public health guidance.

*We are grateful to the reviewer for their considered feedback and suggestions for how our manuscript can be improved.*

While the study is well-written and clearly presented, I have concerns about the methodologic approach. This relates mainly to the assessment (and modelling) of the exposure variables, the highly inter-correlated nature of the two key variables of duration and severity, and the potential for confounding by unadjusted factors. As such, I am concerned about whether the study is providing accurate estimates of the association of obesity duration and the cardiometabolic outcomes. I believe the manuscript could be improved if the authors could address the following comments:

**-	A major concern is residual confounding. Why were there minimal attempts to control for potential confounders - e.g. social-economic status and diet?**

*Response: We believe that variables to be adjusted for should occur prior to the exposure and outcome and thus have the potential to confound. As such, we have adjusted for sex and cohort (and age at follow-up acting as a competing exposure). Additionally, as we are combining three birth cohorts, it is important that any variables that go into the analysis represents the same thing in each cohort (i.e. variables are harmonised across cohorts). Unfortunately, this is only the case for a number of variables in the three cohorts we have used. However, to address this comment we have re-run the analyses additionally adjusting for social class, birth weight, and ethnicity. These variables meet the necessary criteria of 1) being a potential confounder (i.e., potential to cause exposure and outcome) and 2) being measured in each cohort in the same way at the same age. This has necessitated the use of multiple imputation to account for missingness in the covariates. Appropriate detail has been added to the methods section (page 9, lines 279-287).*

**-	How well does the restricted cubic spline model actually capture oscillations or changes in people's BMI given the relatively long (years) interval between measurements? Could the lack of obese 'period' be a function of the way the model is fitted?**

*Response: We agree that the complexity of the trajectories we can fit is limited by relatively small number* and *long intervals of the BMI* measurements. *While we are unable to do* *anything about the measurement schedules of the included cohorts, we aimed to produce as flexible curves as possible with the available data, by modelling using restricted cubic splines, which produce not only good flexibility but also have the benefit of more robust behaviour (than standard cubic splines) at the tails of the distributions. Our aim was to maximise the fit of the models at each measurement occasion. At each stage of model building (location and number of knot points, number of random effects, inclusion of interaction terms etc) we checked model fit statistics (BIC) to make sure improvements in model fit were being made. We have added the following in the limitations (page 16, line 483-485) ‘Our trajectories were dependent on the frequency of BMI measurements across the life course, with some intervals spanning 10 years. As such we may not have captured instances of weight cycling between measurement occasions’.*

**-	How were the categorization cut-off values for duration of obesity chosen?**

*Response: The obesity duration cut-off values were chosen as these split the duration variable into groups which were a compromise between being clinically relevant whilst also maintaining an adequate sample size in each group from which to make robust conclusions. As a sensitivity analyses, we created cut-offs based on quantiles of the obesity duration distribution in order to ensure equal numbers in each category and the pattern of results was consistent.*

**-	Duration and severity very highly correlated - could the authors comment on whether multi-collinearity was an issue and if so how was it dealt with?**

*Response: The nature of the obesity severity variable (i.e. incorporating both duration and extent to which BMI exceeded the BMI cut-off) means it is not particularly surprising that the two were correlated. However, upon checking the variance inflation factor of the obesity severity variable (when entered for adjustment) leads to a VIF at approximately 5.5 in all models, suggesting that collinearity is not a concern. Furthermore, a priori we decided to try and separate the effects of life course duration and life course severity, and thus we specified the model accordingly. It has been shown that when models are correctly specified based on the underlying causal structure that parameter estimates of the effect of interest remained unbiased. This is the case even when there is almost perfect collinearity of the exposure with another variable. It has therefore been concluded that misspecifying the causal structure of the model in order to allay collinearity concerns is much more likely to create bias rather than to be of practical use in avoiding numerical instability. (Schisterman 2017 doi:10.1097/EDE.0000000000000554).*

-	**How accurate are the self-reported BMI measurements? Likely to have differential misreporting by those who are overweight and obese.**

*Response: We have added the following sentence in the limitations section (page 16, line 486-490) ‘ Measurement protocols for weight and height were not consistent within and between studies, which may have introduced bias if, self-reported measurements were systemically under or over-reported. It has been shown that people with greater BMIs tend to under-report their weight [61, 62], suggesting that estimates of obesity duration (and severity) may be conservative in our study.’*

*We did also include a dummy variable for measured vs self-report in the multilevel models, allowing the level-1 residuals to vary depending on whether BMI was measured or self-reported.*

Minor

**-	Additional text in Figure legend 1 would be helpful for readers to interpret the figure.**

*Response: the legend of figure 1 now reads (page 28): ‘Figure 1: Example obesity traits (onset, duration and severity (area-under-the curve and above obesity cut-off)) derived from the BMI-z-score trajectories of two random participants’*

**-	Meaning of line 223-224 unclear: 'data were pooled across cohorts and sexes, enabling adjustment of the association between obesity duration and outcomes for these variables'.**

*Response: We have now reworded this as follows (page 9, line 269-271: Data were pooled across cohorts and sexes, thus enabling adjustment of the association between obesity duration and outcomes cardiometabolic risk factors for cohort and sex’*

**-	Line 265 - remove the word 'people'.**

*Response: Done (page 11, line 321)*

**-	Line 271 - should provide correlation for NCDS too.**

*Response: thank you for picking this up. ‘NCDS: -0.95’ has now been added. (page 1, line 327)*
