# Supplementary material for: Duration of obesity exposure between ages 10 and 40 years and its relationship with cardiometabolic disease risk factors: A cohort study
Source: PLoS Med. 2020 Dec 8;17(12):e1003387. doi: 10.1371/journal.pmed.1003387 (PMC7723271; doi:10.1371/journal.pmed.1003387)
Supplement: Attachment — Submitted filename: Response to reviewer comments_12_09_20.docx [file pmed.1003387.s020.docx]

**Response to reviewer comments_minor revision_12_09_20**

Editorial comments and requests

1. Thank you for noting that the analysis plan was revised based on data that became available and reviewer requests. Please specify exactly how the analyses were changed at each of these points.

*Response*: We have now added this detail, with the resulting text (page 7, lines 193-200), as follows: *“TN and WJ determined which analyses to perform and include in the present paper in January 2019 after discussing options with all co-authors. The analysis plan was revised in May (modelling obesity duration as a categorical variable rather than a continuous variable ) and October 2019 (removing LDL-cholesterol as an outcome due to high amount of missing data) when further exposure and outcome data were obtained and explored. Further analyses were added in June 2020 in response to reviewer comments (adjusting for further putative confounding variables in the regression models, adding sexXduration interaction models in the supplementary analyses)”*

2. Apologies for the confusion, but when I requested the Abstract Conclusions and Author Summary include the potential implications, I thought it might be useful to note potential health policy recommendations could be aimed at reducing early life obesity.

Response: We have now modified these sentences (page 2, line 53 and page 3, line 85) to include the following *“our findings suggest that health policy recommendations aimed at preventing early obesity onset, and therefore reducing lifetime exposure, may help reduce risk of diabetes, independently of obesity severity”.*

3. Thank you for clarifying the ethical approval, please list the institutions and committees that provided this approval.

Response: We have now provided further details to the ethics statement (page 6, lines 167-173), which now reads: *“All of the studies have received ethical approval and obtained informed parental and/or participant consent, both of which cover the secondary analyses reported here. Data collection in the NSHD received multicentre research ethics committee approval (MREC98/1/121), the NCDS obtained ethical approval from the South East MREC (ref:01/1/44) and the BCS70 obtained ethical approval from the National Research Ethics Service (NRES) Committee South East Coast – Brighton and Sussex (Ref. 15/LO/1446). Further details are this information is available from the study websites and/or cohort profiles [27–30].”*

4. Thank you for placing your code in GitHub. Please provide the url in the text.

Response: We have now added the URL to the data availability statement (page 11, lines 301-303), which now reads as follows: *“The statistical code for the analyses in this paper has been placed in GitHub, the open-access online repository (repository url:* [*https://github.com/tomnorris1988/Obesity-duration-and-cardiometabolic-outcomes*](https://github.com/tomnorris1988/Obesity-duration-and-cardiometabolic-outcomes)*”*

5. The Data availability statement does not need to be in the main text, but should be placed in the appropriate metadata field.

Response: We have now removed this.

6. Thank you for including ethnicity data in the Results and Table 1. Please also include the breakdown (49.1% male and 97.2% White British) in the Abstract, and also please note what ethnicities are covered by “other” in a footnote to table 1 (no need to break down numbers of each, though).

Response: We have now added *“(49.1% male and 97.2% White British)”* to the abstract (page 2, line 32) and also added the footnote *“ ^a^other ethnicities: White other, Mixed race, Indian, Pakistani, Bangladeshi, Other Asian, Caribbean, African, Other Black, Chinese”* to table 1 (page 30).

7. While the editors agree that p= 0.05 is an arbitrary cutpoint, it does have the advantage of specificity, while readers may not be clear on what a “weak” association means. Please use more specific language to describe the strength of the associations after adjusting for obesity severity.

Response: Thank you, we apologise if the sentence was ambiguous. In the instance where we describe the association as weak, we have now qualified this by specifically adding *“(p>0.1)”* (page 15, line 397), so the reader knows what we mean by a weak association. In a similar vein, we have also amended the sentence in the discussion which refers to the other study which observed an association in females only, now reading *“though the strength of evidence was modest (p=0.05)”* (page 15, line 427).

PRODUCTION:

1. We have noted that you have the following present in your manuscript: Abbreviations list/ Data Availability Statement

We have removed these section from your manuscript file as this information is automatically pulled from the submission form. Please make sure that the information in the submission form is correct.

*Response: Thank you. We have also now removed these from the current version*

FIGURES and GRAPHICS:

1. Please note that your figures do not currently comply with our publication requirements. For further instructions to assist with your Figure revisions, please refer to our Figure Guidelines at: http://journals.plos.org/plosmedicine/s/figures.

If you need assistance, please contact us directly at graphics@jjeditorial.com so we can further help with this matter. Please send any relevant source files and tell us what application(s) and what operating system you are using.

-Figure 1 has text that looks smaller than 8 points. The legibility of type at sizes smaller than 8 points in the published PDF is frequently compromised. We cannot assume responsibility for the legibility of Figures in the published PDF if there is type smaller than 8 point in them. Please note that this problem will not be detected or corrected by the PACE tool. We recommend that you print figures out to get a better idea of the text legibility. If you are happy with the way they look, we will publish them with the font as is.

Response: We are happy with how the figures look

-Figures 2-4 have been submitted in a layered TIF file format which we don’t accept for Figures. To ensure that your Figures publish properly, please flatten the layers in each Figure file (Layer→Flatten Image in Photoshop, Image→Flatten Image in GIMP) and re-upload. Make sure to save your TIF files with LZW Compression turned on.

Response: We have now flattened the figures

-Figures 2-4 currently contain more than ½ an inch of white space on any side. Please remove unnecessary white space, leaving about ⅛ inch of white space outside of the image. Once you have made your selection, go to Image → Crop to Selection. After cropping, always be sure to check the size.

Response: We have now cropped the figures to remove excess white space at the top and left of figures 2-4

-Figures 2-4 exceed the dimension limits for Figures. Figures must be less than 19.05cm in width, and less than 22.225cm in height. Please note that figures will be typeset at the dimensions provided. Please make the necessary adjustments and re-upload your Figures. In Photoshop: Image→Image Size. Make sure the Resample Image checkbox is NOT checked. If the Width is over 19.05cm, type 19.05 in the Width box (19.05cm is our maximum allowable width for figures). In GIMP: Image→Scale Image. Set the units of measurement, in the pull down menu next to Height, to millimeters. If the Width is over 190.5mm, type 190.5 in the Width box (19.05cm is our maximum allowable width for figures) and hit Tab. The new Height of the figure will appear, scaled proportionately to the change in Width.

While revising figures 2-4 , please keep in mind that we ask that all text be between 8-12 point font (2.8 - 4.2 mm). It is still possible to read 5-6 point type but should only be used sparingly in your figure. For example, it is acceptable for numbers along an axis of a graph or for subscripts and superscripts. 4 point, or smaller, text is not allowed. We recommend that you print figures out to get a better idea of the text legibility.

-Figures 2-4 are low resolution and will appear blurry or pixelated if published. Text and images should appear crisp and clear. Please note that this problem will not be detected or corrected by the PACE tool, and this will not be fixed by simply increasing the resolution of your Figures in the image editing software. Please carefully review the PLoS Figure Guidelines and provide Figure files that were originally created at 300 pixels/inch (dpi) and at least 6.68cm wide, but less than 19.05cm and under 22.225cm tall. For further information, please see the Figure Guidelines (http://journals.plos.org/plosmedicine/s/figures).

1) Export a PDF from PowerPoint/Word/Excel (or other image source file). On a Mac, click on the 'PDF' button in the printer dialog box and select 'Save as PDF'. On a PC, send the file to print and select 'Adobe PDF' or 'PDFCreator' (it's freeware) from your list of available printers.

If you are using Adobe PDF, then go to Printer Properties, Default Settings and select Press Quality.

If you are using PDFCreator, Go to 'Printer Properties' and then click on 'Advanced' on the bottom right. Under 'Graphic', go to 'Print Quality' and select 600dpi.

2) PHOTOSHOP: When you open up the PDF, make sure that the 'Constrain Proportions' box is checked. Set the 'Resolution' to 300dpi (118.11 pixels/cm) and set the 'Bit Depth' to 8 bit. Use the crop tool (next to the eyedropper) to remove unnecessary white space. Go to - Image - Image Size. Make sure the 'Constrain Proportions' and 'Resample Image” boxes are checked.

To ensure that your Figures publish properly, please delete the alpha channels from each Figure file. In Photoshop, select it in the Channels Palette and click on the trash can icon. When you save the TIFFs make sure that the 'LZW' button is checked and 'Discard Layers and Save a Copy' button is checked.

3) GIMP: When you open up the PDF, set the resolution to 300. Make sure the chain link icon is joined together. Then change the size of your figure according to the pixel specifications mentioned in step 2) above. To ensure that your Figures publish properly, please delete the alpha channels from each Figure file. In GIMP, go to Layers - Transparency - Remove Alpha Channel. When you are done, 'SAVE AS' (or Export if GIMP 2.8). Select File Type - 1st TIFF option. Make sure to use LZW compression.

4) Print out your figures on a desktop printer. Text and graphics should appear clear and crisp with no pixelated or blurred edges.

5) If the resulting figures are still blurry, then your original source images are too low of a resolution. Please increase the resolution of your original source images, and repeat this process.

Response: We have followed the above steps and hope that the figures are now the appropriate format.
